# Medication Overuse Withdrawal in Children and Adolescents Does Not Always Improve Headache: A Cross-Sectional Study

**DOI:** 10.3389/fneur.2020.00823

**Published:** 2020-08-19

**Authors:** Romina Moavero, Maddalena Stornelli, Laura Papetti, Fabiana Ursitti, Michela Ada Noris Ferilli, Martina Balestri, Giorgia Sforza, Samuela Tarantino, Federico Vigevano, Massimiliano Valeriani

**Affiliations:** ^1^Child Neurology Unit, Neuroscience and Neurorehabilitation Department, Headache Center, Bambino Gesù Children's Hospital, IRCCS, Rome, Italy; ^2^Child Neurology and Psychiatry Unit, Tor Vergata University of Rome, Rome, Italy; ^3^Center for Sensory Motor Interaction, Aalborg University, Aalborg, Denmark

**Keywords:** chronic migraine, medication overuse headache, children, ICHD-3 criteria, secondary headache, treatment

## Abstract

**Background:** MOH can be diagnosed in subjects with headache occurring 15 days/month in association with a regular medication overuse, but its existence is not universally accepted. ICHD-3 redefined criteria for MOH, removing the criterion associating drug suspension with headache course. The aim of our study was to compare the rate of patients diagnosed with medication overuse headache (MOH) according to ICHD-2 and ICHD-3 criteria, to verify the degree of concordance. The secondary aim was to verify if drug withdrawal was really associated with pain relief.

**Methods:** In this cross-sectional study, we retrospectively analyzed a sample of 400 patients followed for primary chronic headache at the Headache Center of Bambino Gesù Children's Hospital. We then selected those presenting with a history of medication overuse, and we applied both ICHD-2 and ICHD-3 criteria to verify in which patients the criteria would identify a clinical diagnosis of MOH.

**Results:** We identified 42 subjects (10.5%) with MOH; 23 of them (55%) presented a relief of headache withdrawing drug overuse. Regarding the applicability of the ICHD-2 criteria, 43% of patients (18/42) fulfilled all criteria, while all ICHD-3 diagnostic criteria were satisfied in 76% of patients (32/42). Eighteen patients (43%) satisfied both ICHD-2 and ICHD-3 criteria, while 10 patients (24%) did not satisfy either diagnostic criterion.

**Conclusions:** Our study suggests that in children and adolescents, withdrawing medication overuse is not always associated with a clinical benefit. Therefore, though allowing a MOH diagnosis in a higher rate of patients as compared to ICHD-2, the application of ICHD-3 criteria does not guarantee a true a causal relationship between medication overuse and headache worsening.

## Introduction

Medication overuse headache (MOH) is a headache occurring on 15 or more days/month in a patient with a preexisting primary headache and developing as a consequence of regular overuse of acute or symptomatic headache medication (on 10 or more or 15 or more days/month, depending on the medication) for more than 3 months (1). MOH is listed as a secondary headache, in the section focused on “Headache attributed to a substance or its withdrawal.”

Although pathophysiologic mechanisms of MOH are still largely unclear, a genetic predisposition likely plays an important role (2, 3). Another potentially significant pathogenetic factor taken into consideration is the interaction between drugs used and neurotransmitters (4) and/or hormonal systems (5). Other factors investigated over time include the presence of abnormal neuronal excitability (6) and changes in gray matter volumes (7) and cerebral metabolism (8–10).

The overall prevalence of MOH in the general population is 0.5–2.6%, although it varies between different studies, probably as a consequence of different diagnostic criteria published over time and different methods used to collect epidemiological data (10, 11). Very few epidemiological studies are available in the pediatric population. Data from Norway and Taiwan report prevalence rate of 0.2 and 0.3%, respectively (12, 13). Data from pediatric populations with chronic primary headache disorders report a medication overuse in 10–60% of cases (14). Both in adults and in children, MOH appears to be more common among females than among males (15, 16). Hopefully, a planned study will clarify some aspect of pediatric MOH (17). This trial plans to evaluate whether the frequency of acute medication overuse is associated with headache frequency in children and adolescents, and the outcomes will be frequency of headache, change in headache frequency in relation to use of acute medications, and headache-related disability (17).

MOH clinical features are usually the same of preexisting primary headache disorder (10). In pediatric patients, it is more commonly associated with chronic migraine (CM) (18). Non-steroid anti-inflammatory drugs (NSAIDs) are the class of drugs more often overused, followed by paracetamol and triptans (15). Historically, the treatment of MOH includes two main strategies: a detoxification program with discontinuation of drugs overused and initiation of pharmacological and non-pharmacological preventive therapy (10).

In the last two decades, diagnostic criteria for MOH were gradually changed. Initially, MOH could be diagnosed only if the headache resolved or reverted to the previous pattern within 2 months after withdrawal of the overused medication (19). In the revision of diagnostic criteria published in 2006 (20), the Headache Classification Committee proposed to remove the criterion concerning the effect of drug suspension on headache course, and this modification was kept in the last published version of ICHD-3 (Table 1) (1).

**Table 1:** Diagnostic criteria for MOH by ICHD-2 (2004) [(35) and by ICHD-3 (1)].

**ICHD-2**	**ICHD-3**
**A**. Headache present on ≥15 days/month fulfilling criteria C and D **B**. Regular overuse for ≥3 months of one or more drugs that can be taken for acute and/or symptomatic treatment of headache **C**. Headache has developed or markedly worsened during medication overuse **D**. Headache resolves or reverts to its previous pattern within 2 months after discontinuation of overused medication	**A**. Headache occurring on ≥15 days/month in a patient with a preexisting headache disorder **B**. Regular overuse for >3 months of one or more drugs that can be taken for acute and/or symptomatic treatment of headache **C**. Not better accounted for by another ICHD-3 diagnosis

Therefore, MOH can be presently diagnosed in a subject with a history of a preexisting primary headache, presenting with headache occurring 15 days per month in association with a regular medication use exceeding specific thresholds.

A direct consequence of new criteria could be an increase in definite diagnosis, since MOH can now be diagnosed even in the absence of improvement after drug withdrawal. However, diagnostic criteria and even the existence of this specific nosographic entity are not universally accepted. For instance, some authors wondered whether medication overuse is the real cause of headache in all subjects fulfilling diagnostic criteria for MOH (14, 19, 21). Indeed, in some individuals medication overuse can increase headache frequency, and discontinuing the medications can have a benefit, but this is not the case in all individuals overusing medications. In some case, increasing headache frequency represents a worsening of the primary headache disorder, and increased use of acute medications is its consequence (14).

The aim of our study was to compare the rate of patients diagnosed with MOH according to the old ICHD-2 and new ICHD-3 criteria, in order to verify the degree of concordance and understand if the new classification really led to different diagnostic rates. The secondary aim was to verify if drug withdrawal is really associated with pain relief and therefore to investigate in a large sample of pediatric patients whether MOH is a true entity.

## Materials and Methods

In this cross-sectional study, we retrospectively analyzed a sample of patients followed at the Headache Center of the Neuroscience Department of Bambino Gesù Pediatric Hospital in Rome. We included all patients with chronic headache, diagnosed according to the ICHD-3 criteria (1), and followed up at our Headache Center in the period 2010–2018, whose parents gave their informed consent to be contacted for retrospective studies. The sample was partially published in Papetti et al. (18). In particular, 210 out of 377 patients included in the Papetti et al.'s sample (collected between 2010 and 2016) were considered for the present study while the remaining 190 patients were totally original. Moreover, only 20 out 42 of the MOH patients were issued from the Papetti et al. population, while the remaining 22 patients are totally original. As compared to Papetti et al., the present study investigated different points: (1) the comparison of the applicability of the ICHD-2 and ICHD-3 criteria of pediatric MOH patients and (2) the clinical outcome after medication withdrawal in MOH children and adolescents.

Among these patients, we selected those presenting with a personal history of medication overuse, defined as regular use of abortive therapy: at least 10 days per month for ergotamine, triptans, opioids, or combination-analgesic medication and 15 or more days per month for non-opioid analgesics (paracetamol, non-steroidal anti-inflammatory drug, or acetylsalicylic acid). Overuse should have been carried on for at least 3 months. In all patients, the clinical diagnosis of MOH was tested according to either ICHD-2 or ICHD-3 version criteria, in order to verify the degree of concordance. The diagnoses were made independently by two experienced neurologists, blinded to each other's rating (MV, LP). All these data were initially extrapolated by clinical charts and then confirmed and deeply investigated during follow-up visits and/or telephonic interviews.

Clinical data collected for each patient were the preexisting primary headache type, the clinical characteristics of headache and other symptoms associated, and the treatment used, both symptomatic and prophylactic.

The usual therapeutic strategy was an intensive verbal advice to discontinue the medication overuse, with the suggestion of a different symptomatic treatment than the overused one. In almost all cases, a preventive medication was also proposed, at this same time. Medication withdrawal was considered successful if criteria for overuse were no more satisfied, and it was conducted over a 2-months period. The outcome of medication withdrawal was assessed after two additional months of follow-up, and it was considered effective if chronic headache reverted to episodic.

Ethical Board approval for retrospective study was obtained.

### Statistical Analysis

Statistical analysis was conducted by SPSS version 22.0. To test the hypothesis of a possible association between response to medication withdrawal and sex, type of overused medication, and preventive treatment, we used the χ^2^ test. A *p*-value of ≤0.05 was considered significant.

Furthermore, a multiple-regression logistic analysis has been used to evaluate whether age, age at first attack (0–6, 7–10, 11–14, 15–18 years), or type of preventive treatment (topiramate, 5-hydroxytryptophan, flunarizine, amitriptyline) influenced response to withdrawing overused medication. Response to medication withdrawal was selected as a dependent variable, and then all the other variables have been tested as independent variables in a block entry to evaluate the *t* value, the significance, the standard error, and the upper and lower limit in a confidence interval of 95%.

## Results

**Figure 1:**
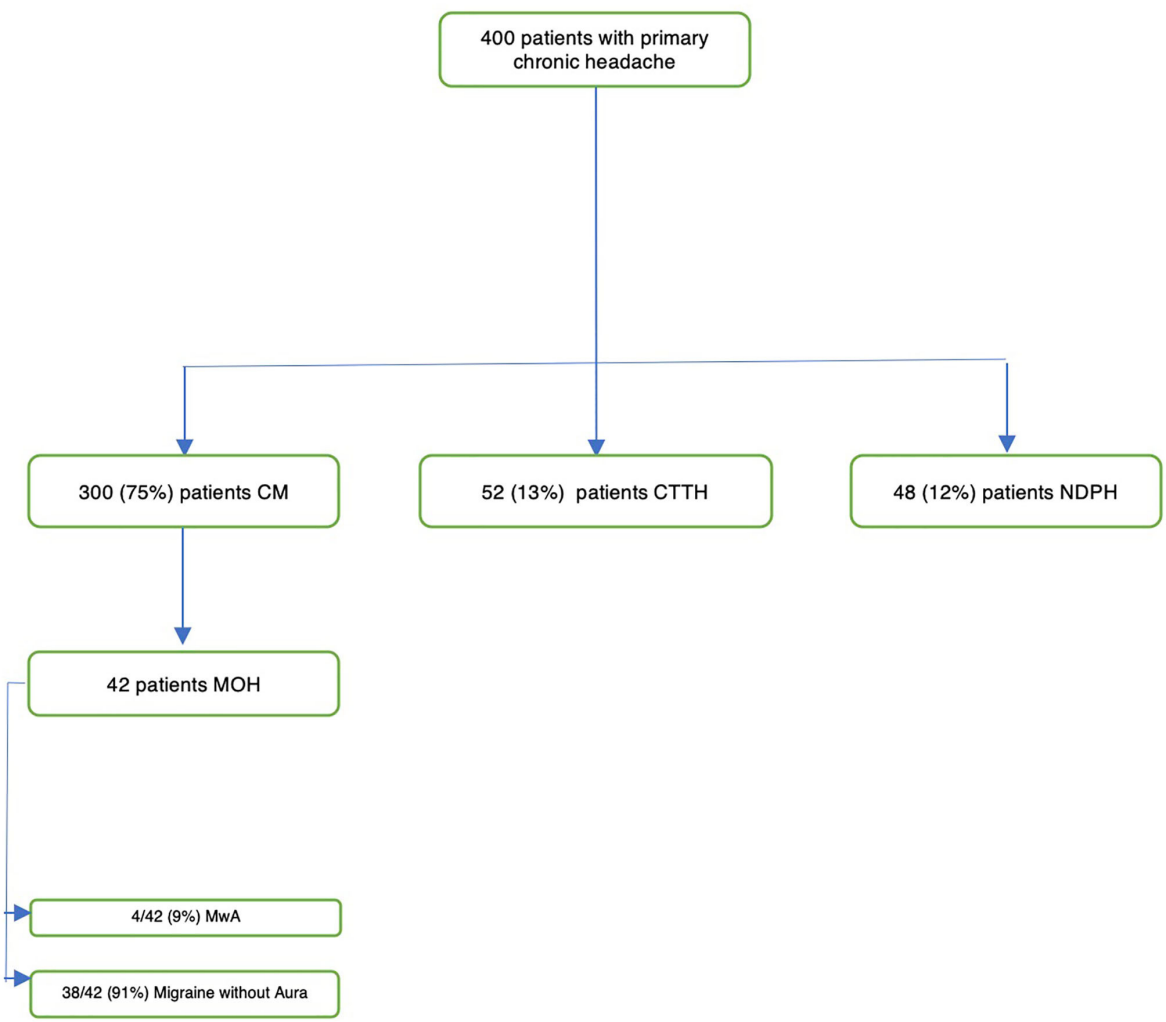
Flowchart showing patients' disposition.

We collected and analyzed clinical data from a sample of 400 patients (134 M, 266 F) with primary chronic headache. There were no missing data in our sample. Seventy-five percent of patients presented with CM, 13% with chronic tension-type headache, and 12% with new daily persistent headache (NDPH) (Figure 1). In 11% of patients (10 patients with NDPH and 37 with CM), migraine with aura (Mwa) was diagnosed.

In this sample, we identified 42 subjects (10.5%, Table 2) with symptomatic medication overuse defined as above (at least 10 days per month for ergotamine, triptans, and opioids and 15 or more days per month for non-opioid analgesics). The sample was mainly composed of females (11 M, 26%−31 F, 74%), with a mean age of 13 years at their first medical examination (range: 8–17 years). All patients (100%) presented CM, and 9% (4/42) presented also Mwa. The age at onset of headache was ≤6 years in 9% of patients (4/42), 7–10 years in 29% (12/42), 11–14 years in 48% (20/42), and 15–18 in 14% (6/42). The mean duration of medication overuse was 4.1 months (range 3–6 months).

**Table 2:** Demographic features of patients with chronic migraine and medication overuse.

	***N***	**%**
**Patients**	42	100
Mean age: 13 years (range 8–17 years)	–	–
**Sex**		
•Males	11	26
•Females	31	74
**Diagnosis**		
•Chronic migraine	42	100
•Migraine with aura	4	9
**Age at onset**		
• <6 years	4	9
•7–10 years	12	29
•11–14 years	20	48
•15–18 years	6	14
**Symptoms associated**		
•Photophobia	34	81
•Phonophobia	34	81
•Nausea and vomit	30	71
•Dizziness	18	42
**Symptomatic treatment**	42	100
•NSAIDs	42	100
•Triptans	9	21
**Prophylactic treatment**	39	93
•Amitriptyline	31	79
•Topiramate	15	38
•Flunarizine	11	28
•Tryptophan	6	15

Photophobia and phonophobia were both present in 81% of patients (34/42), nausea and vomiting in 71% (30/42), and dizziness in 42% (18/42). All patients used NSAIDs as symptomatic treatment; 21% of the sample (9/42) used triptans as further option after a poor response to NSAIDs. Moreover, prophylactic treatment was prescribed in 93% (39/42) of patients, including drug-naïve patients and those who were assuming an ineffective prophylactic therapy. Amitriptyline was the most used drug (79%, 33/42); topiramate was used in 38% (16/42), flunarizine in 28% (12/42), and tryptophan in 15% (6/42). More than one type of prophylactic drug was used in 28% of the sample (12/42; these patients were already assuming one prophylactic drug at the time of our first visit). After withdrawing symptomatic drug overuse, a clear benefit was evident only in 23/42 subjects (55%).

**Figure 2:**
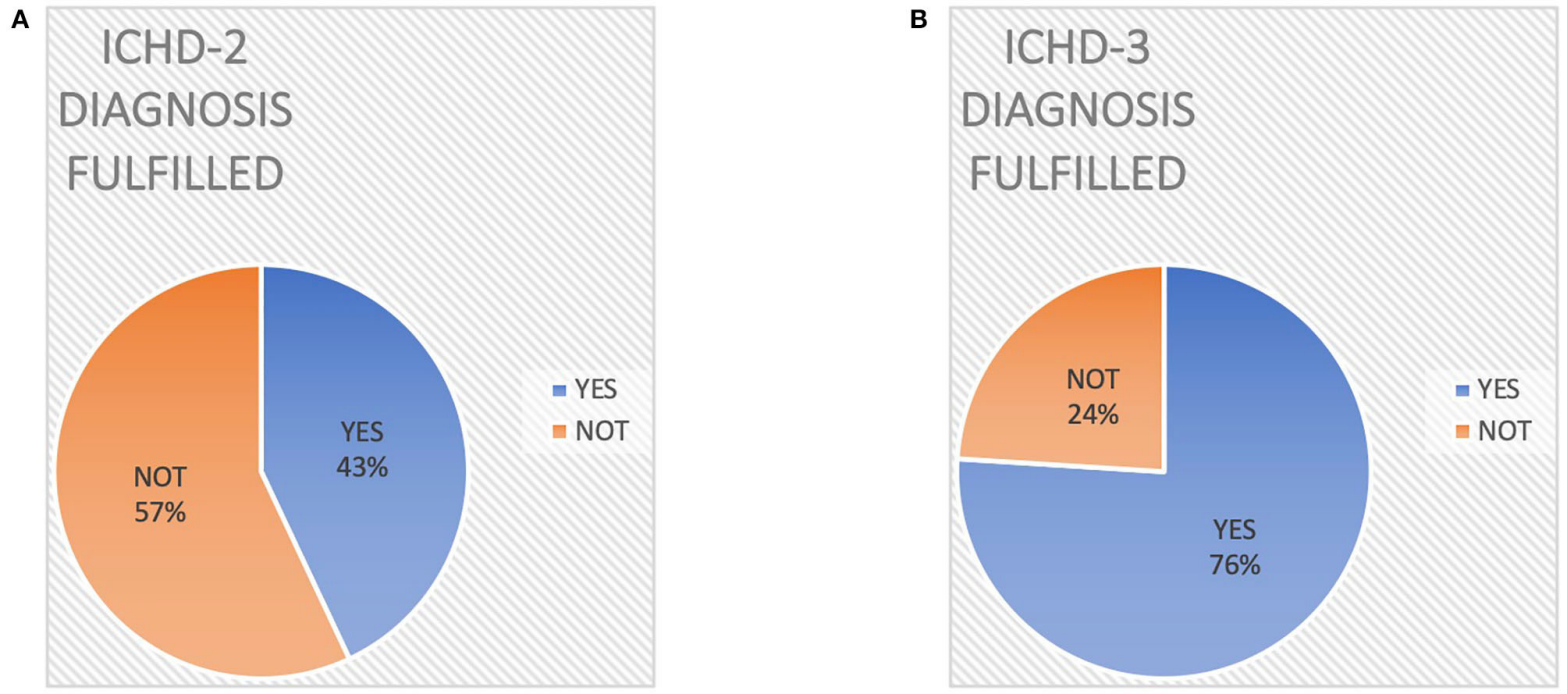
Different diagnostic rate of Medication Overuse Headache according to ICHD-2 criteria **(A)** vs. ICHD-3 criteria **(B)**.

**Figure 3:**
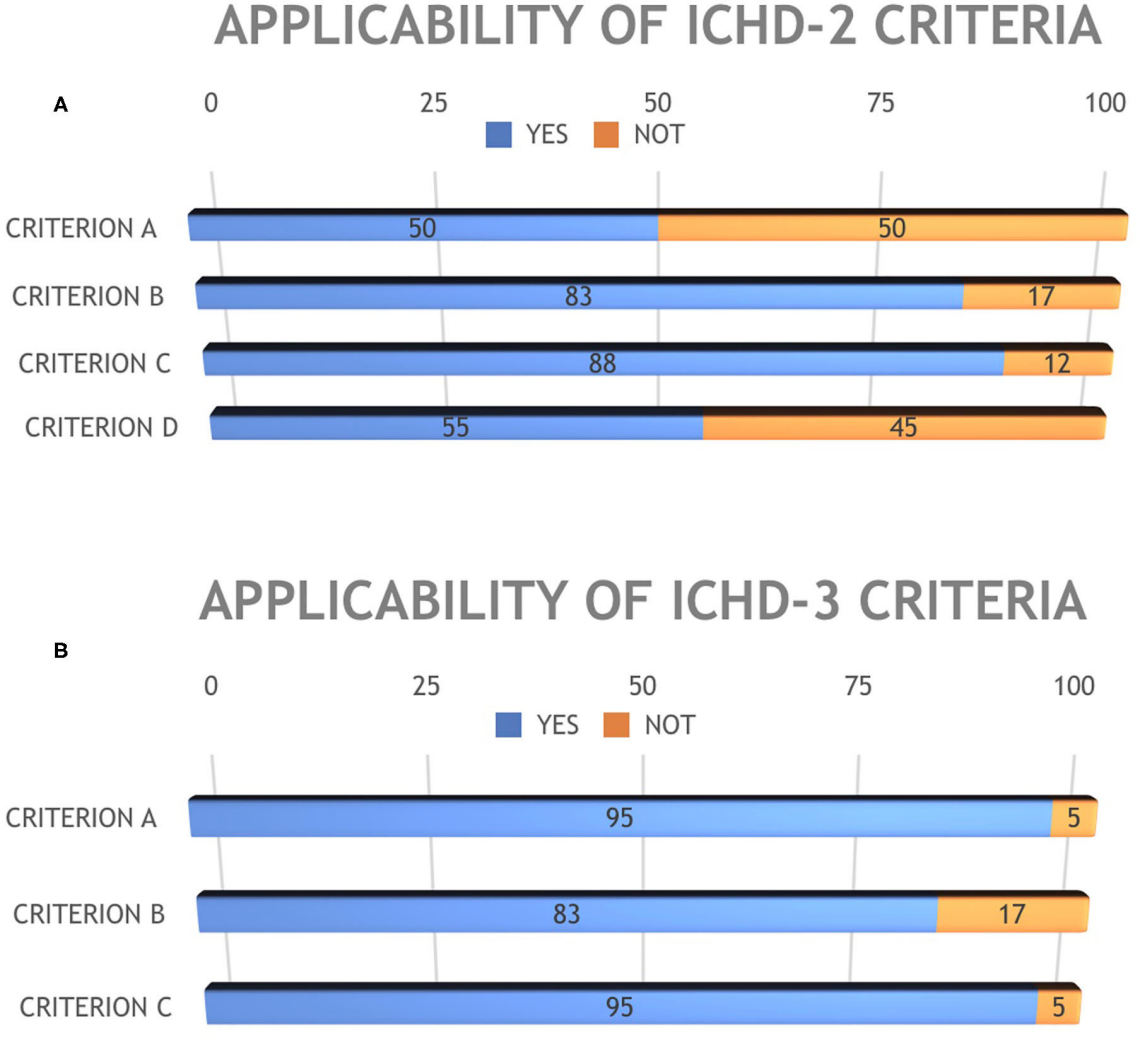
Applicability of ICHD-2 **(A)** and ICHD-3 **(B)** diagnostic criteria for MOH.

Regarding the applicability of the ICHD-2 criteria, 43% of patients (18/42) fulfilled the diagnosis of MOH while 57% (24/42) did not fulfill all the diagnostic criteria (Figure 2). In detail, 21/42 patients (50%) fulfilled criterion A; 35/42 (83%) criterion B, 37/42 (88%) criterion C, and 23/42 (55%) criterion D (Figure 3A).

On the other hand, all ICHD-3 diagnostic criteria were fulfilled in 76% of patients (32/42, Figure 1). Specifically, ICHD-3 criterion A was fulfilled by 40/42 patients (95%), criterion B by 35/42 (83%), and criterion C by 40/42 (95%) (Figure 3B).

Eighteen patients (43%) satisfied both ICHD-2 and ICHD-3 criteria, while 10 patients (24%) did not satisfy either diagnostic criterion.

None of the analyzed variables (age at evaluation, age at first attack, or type of preventive treatment) showed a statistical significance at the multivariate analysis (Table 3). The improvement after drug overuse withdrawal was observed in 20/31 (65%) of the females of our sample, compared with 3/8 (38%) of males (*p* = 0.03). However, we have to underline that our sample was mainly composed of females. The type of overused drug was not associated with response to withdrawal (*p* = 0.93).

**Table 3:** Results of multiple-regression logistic analysis: beta coefficients and significance, with lower and upper limits at 95% confidence interval.

	**β coefficient ± standard error**	**Significance**	**Confidence interval (95%)**
Age	0.001 ± 0.004	0.882	−0.007; 0.009
Age at migraine onset 0–6 years	−0.483 ± 0.375	0.207	−1.246; 0.280
Age at migraine onset 7–10 years	−0.246 ± 0.232	0.295	−0.718; 0.225
Age at migraine onset 11–14 years	−0.022 ± 0.155	0.213	−0.117; 0.508
Age at migraine onset 15–18 years	−0.005 ± 0.262	0.934	−0.554; 0.511
Topiramate	−0.005 ± 0.207	0.981	−0.427; 0.416
Flunarizine	0.065 ± 0.236	0.785	−0.415; 0.545
TriptOH	−0.339 ± 0.273	0.223	−0.895; 0.217
Amitriptyline	0.179 ± 0.201	0.379	−0.229; 0.587

## Discussion

Our retrospective study on a large sample of pediatric patients revealed that the application of ICHD-3 criteria allows a MOH diagnosis in a higher rate of patients (76 vs. 43%), thus proving more sensitive than ICHD-2 criteria. The main difference between the two versions is that ICHD-3 criteria do not require remission or improvement of headache after the regular drugs overuse is stopped. However, ICHD-3 version, removing the relationship between pain and drug overuse, seems to consider the MOH as a fully established diagnosis, while it is still a matter of debate. In ICHD-2, only 50% of patients satisfied criterion A, since many patients did not satisfy criteria C and D. As for ICHD-3, the two patients not satisfying this criterion were adolescents who received the first diagnosis of migraine after initiating the abuse. In both versions of ICHD, only 83% of patients satisfied criterion B, since the remaining 17% of patients presented an overuse of medication for <3 months. Finally, two patients did not satisfy ICHD-3 criterion C since after a careful examination of data it was doubtful if they could be classified as “Headache attributed to non-vascular intracranial disorders.”

A second crucial finding of our study is that in our sample symptomatic drug withdrawal was not always sufficient to revert chronic to episodic migraine, thus strengthening the concept that, in turn, medication overuse was probably not sufficient to make our patients' migraine become chronic. Specifically, in our sample, medication withdrawal did not cause any reduction in headache frequency in almost half of patients (45%). Furthermore, 22/23 patients (95%) showing an improvement of symptoms after drug withdrawal (meaning a return to episodic headache) were assuming a preventive therapy at the same time. Therefore, it is very difficult to judge if the positive effect on headache frequency was caused by one or the other therapeutic approach used.

The few studies published on MOH in pediatric age show a response rate to drug withdrawal (defined as a reduction more than 50% of headache frequency) between 40 and 77% (2, 22–24). On the other hand, a lack of improvement after drug withdrawal is reported in 4–41% of patients (Table 3). A genetic study on a pediatric population with CM and medication overuse identified statistically significant gene expression differences between responders and non-responders to withdrawal, thus suggesting a possible biomarker to distinguish true MOH patients from chronic migraineurs in whom overused medication does not have a pathophysiological role (2).

Considering also MOH studies in adults, we found limited evidence supporting a clear benefit of discontinuation of symptomatic medications without concomitant introduction of a preventive therapy (25). In particular, clear clinical benefits after only withdrawing overused medication have been described in less than one third of reported patients (26–28). Another important bias of the available studies is represented by patients who pretend to have withdrawn symptomatic treatment, while keeping overusing drugs. Furthermore, randomized controlled clinical trial investigating topiramate (29, 30) and onabotulinumtoxinA (31, 32) for treatment of CM showed that immediate initiation of preventive treatment without early suspension of the overused medication is effective in patients with CM and medication overuse (33). Most of these studies also lacked an adequate control group, thus making it impossible to differentiate patients presenting a benefit due to the typical cyclic pattern of headache, and those really responding to overuse cessation. Some authors hypothesized that medication overuse can be seen as an epiphenomenon of a chronic headache presenting with periods of higher frequency and severity (19), thus suggesting that a combined strategy of preventive therapy and overuse cessation could be more appropriate. Indeed, a recent review of the available literature data concluded that the combined approach of discontinuation of overused acute medications and a concurrent preventive intervention should be the standard of care (25), as already recommended by EFNS (European Federation of Neurological Societies) guidelines for MOH (34).

### Limitations of the Study

Our study certainly presents some limitations. First of all, the retrospective nature of the study is a limitation in itself. Furthermore, our population might not be representative of the general population, as patients have been recruited in a tertiary headache center. However, it is also important to underline that patients suffering from chronic headache and medication overuse usually refer to tertiary centers, and therefore, our sample might be overlapping to general pediatric MOH samples. Furthermore, some patients could present with comorbidities, such as obesity, anxiety, and depression, which could influence the outcome but that have not been taken into consideration in the present analysis. Lastly, the information of drug use is based on patients' diary, and especially in case of adolescents, these data might not always be completely reliable.

## Conclusions

In conclusion, our data on a large pediatric population of subjects with chronic headache and medication overuse show that withdrawing medication overuse is not always associated with a clinical benefit. This means that a causal relationship between medication overuse and headache worsening is not always demonstrable, thus suggesting that the concept of MOH might be not universally applicable. Although ICHD-3 criteria for MOH appear to be more sensitive than ICHD-2, allowing a definite diagnosis in a higher number of patients, they do not contribute to make this issue less puzzling, since the new ICHD version considers MOH as a definite nosographic entity, which is not supported by the present literature. In other words, if the effect of drug suspension on headache course is not verified, a sure relationship between medication overuse and headache chronification cannot be demonstrated in all patients. A proposal for a new systematic review on pediatric MOH has been recently published (17) and will hopefully contribute to clarify this issue.

## Data Availability Statement

The data analyzed in this study is subject to the following licenses/restrictions: Privacy. Requests to access these datasets should be directed to romina.moavero@opbg.net.

## Ethics Statement

The studies involving human participants were reviewed and approved by Bambino Gesù Ethical Board. Written informed consent to participate in this study was provided by the participants' legal guardian/next of kin.

## Author Contributions

RM, LP, and MV conceptualized the study. RM, LP, and MS inserted data in the database and analyzed the data. LP, MS, FU, MF, MB, GS, and MV followed up all the patients included in the study. RM drafted the manuscript. FV and MV revised the different versions of the manuscript. All the authors revised and approved the final version of the manuscript. All authors contributed to the article and approved the submitted version.

## Conflict of Interest

The authors declare that the research was conducted in the absence of any commercial or financial relationships that could be construed as a potential conflict of interest.
